# Graphene Oxide Quantum Dots Derived from Coal for Bioimaging: Facile and Green Approach

**DOI:** 10.1038/s41598-018-37479-6

**Published:** 2019-03-11

**Authors:** Sukhyun Kang, Kang Min Kim, Kyunghwan jung, Yong Son, Sungwook Mhin, Jeong Ho Ryu, Kwang Bo Shim, Byoungsoo Lee, HyukSu Han, Taeseup Song

**Affiliations:** 1 Korea Institute of Industrial Technology, School of Materials, Science and Engineering, Hongik University, Sejong, 339-701 Republic of Korea; 2 Department of Materials Science and Engineering, Hanyang University, Hanyang Universiy, Seoul, 133-791 South Korea; 3 Korea Institute of Industrial Technology, 113-58, Seohaean-ro, Siheung-si, Gyeonggi-do, 15014 Republic of Korea; 4 Department of Materials Science and Engineering, Korea National University of Transportation 50 Daehak-ro, Chungju-si, Chungbuk 380-702 Republic of Korea; 5 Department of Energy Engineering, Hanyang Universiy, Seoul, 133-791 South Korea

## Abstract

Graphene oxide quantum dots (GOQDs) are usually prepared using expensive carbon precursors such as carbon nanotubes (CNT) or graphene under the strong acidic condition, which requires an additional purifying process. Here, we first develop a facile pulsed laser ablation in liquid (PLAL) technique for preparing GOQDs using earth-abundant and low-cost coal as a precursor. Only ethanol and coal are used to produce GOQDs with excellent optical properties. The prepared GOQDs exhibit excellent optoelectronic properties which can be successfully utilized in bioimaging applications.

## Introduction

Graphene oxide (GO), with superior properties that are suited to a broad range of applications, including optoelectronic and biological applications, has gained considerable attention^1–12^. The optical properties and hydrophilicity of GO can be tuned by downsizing GO particles to the few-nanometer scale; these particles are referred to as “graphene oxide quantum dots” (GOQDs)^13–21^. GOQDs are promising candidates for biomedical applications due to their lack of toxicity as well as their hydrophilicity and high light-emitting efficiency that originate from quantum-confinement and edge effects associated with their oxygen-containing functional groups^16^.

Coal is one of the most Earth-abundant and low-cost sources of carbon that can be used for the preparation of GOQDs (Supplementary Tables S1–2. Wet-chemical syntheses of GOQDs generally result in acidic residues that require additional purification steps in order to obtain GOQDs^14,15^. Moreover, these residues modify the physicochemical properties of GOQD functional groups, resulting in poor optoelectronic properties. As a result, expensive processing as well as poor optoelectronic properties limit the practical use of coal as a carbon source for the preparation of GOQDs through wet-chemical routes.

The synthesis of nanomaterials by pulsed laser ablation in liquid (PLAL) has attracted significant levels of attention^22–28^. PLAL is capable of downsizing bulk particles into few-nanometer-sized QDs within a few minutes. At the same time, PLAL does not require the use of strongly acidic (e.g. KMnO_4_, H_2_SO_4_) or basic aqueous solutions (e.g. KOH, HNO_3_)^12–16,19^. Hence, PLAL can be used to synthesize low-cost and high-quality GOQDs. Herein, we report the first facile and environmentally friendly PLAL method for the fabrication of GOQDs from coal. Ethanol and coal are the only reactants used in this method. The as-prepared GOQDs exhibit diameters that range from 5 to 30 nm. The entire process requires only 5 min, and no purification steps are required. The GOQDs prepared in this manner are highly photostable and crystalline. The GOQDs also exhibit low toxicity and excellent biocompatibility, making them promising materials for bioimaging applications. Finally, we successfully demonstrated the practical use of these GOQDs as high-performance photoluminescence (PL) probes by bioimaging Pancreas cancer cells (PanC-1).

## Results and Discussion

GOQDs were fabricated by the simple and facile PLAL process using coal in high-purity ethanol. In a typical process, 1 g of coal (purity of up to 90%) was dispersed in 30 mL of high-purity ethanol. The concentration of the coal suspension was about 0.03 g mL^−1^. A Q-switch ND:YAG laser system was employed at room temperature and in the air. The coal suspension, which forms a vertical water column, was ablated by a horizontal pulsed laser beam (355 nm, third harmonic, 10 ns pulse width) at a repetition rate of 10 Hz. The pulsed laser beam was focused to a diameter of around 1 mm with an ablation energy of 0.1 J.

**Figure 1:**
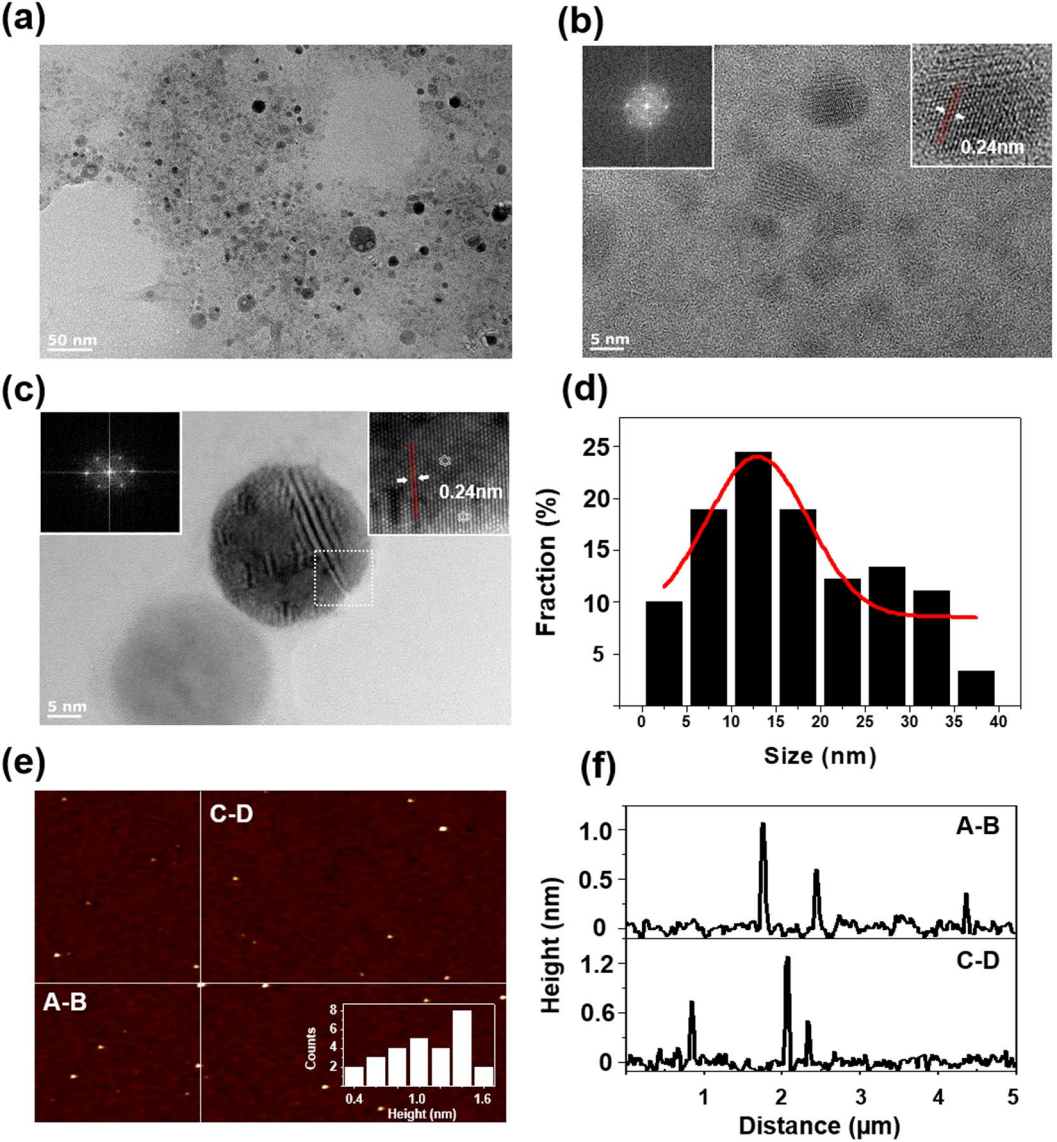
TEM and AFM characterization of GOQDs. (**a**) TEM images of GOQDs. (**b**,**c**) HR TEM images of GOQDs, insets are the 2D FFT patterns (left). (**d**) the size distribution of GOQDs. (**e**) AFM image of GOQDs and (**f**) the height profile of GOQDs.

Transmission electron microscopy (TEM) revealed that coal particles transformed into GOQDs after PLAL processing for 5 min (Fig. 1a). GOQDs with distinct crystal structures were observed by high-resolution TEM (HR-TEM, Fig. 1b,c). A 0.24-nm lattice fringe corresponding to the [1120] plane was clearly evident in the HR-TEM image of the GOQDs. No crystalline features that correspond to graphite, such as its [002] plane, were observed by HR-TEM^29–32^. About 101 particles were investigated to evaluate average diameter. The size distribution of the GOQDs was fitted by a Gaussian curve with 95% confidence interval (Fig. 1d). The result revealed an average diameter of 20 ± 10.25 nm of the GOQDs. In addition, atomic force microscopy (AFM) revealed GOQD heights of 0.4–1.6 nm, indicating that the as-prepared GOQDs consist of only single or few-layer graphene sheets (Fig. 1e,f)^15,29^. As prepared GOQDs were centrifuged at 12000 rpm for 30 min to separate any impurity suspended in solution. The centrifuged solution was filtered through syringe filters (Millipore, 0.22 mm pore size). After that, the GOQDs were dried overnight in vacuum oven at 80 °C and their gross weights were weighed. The yield was calculated as about 18% where the weight of the dried GOQDs product was divided by the weight of the starting material.These results demonstrate that coal can be completely transformed into GOQDs by the facile, low-cost, and environmentally friendly PLAL technique at room temperature within a few minutes.

**Figure 2:**
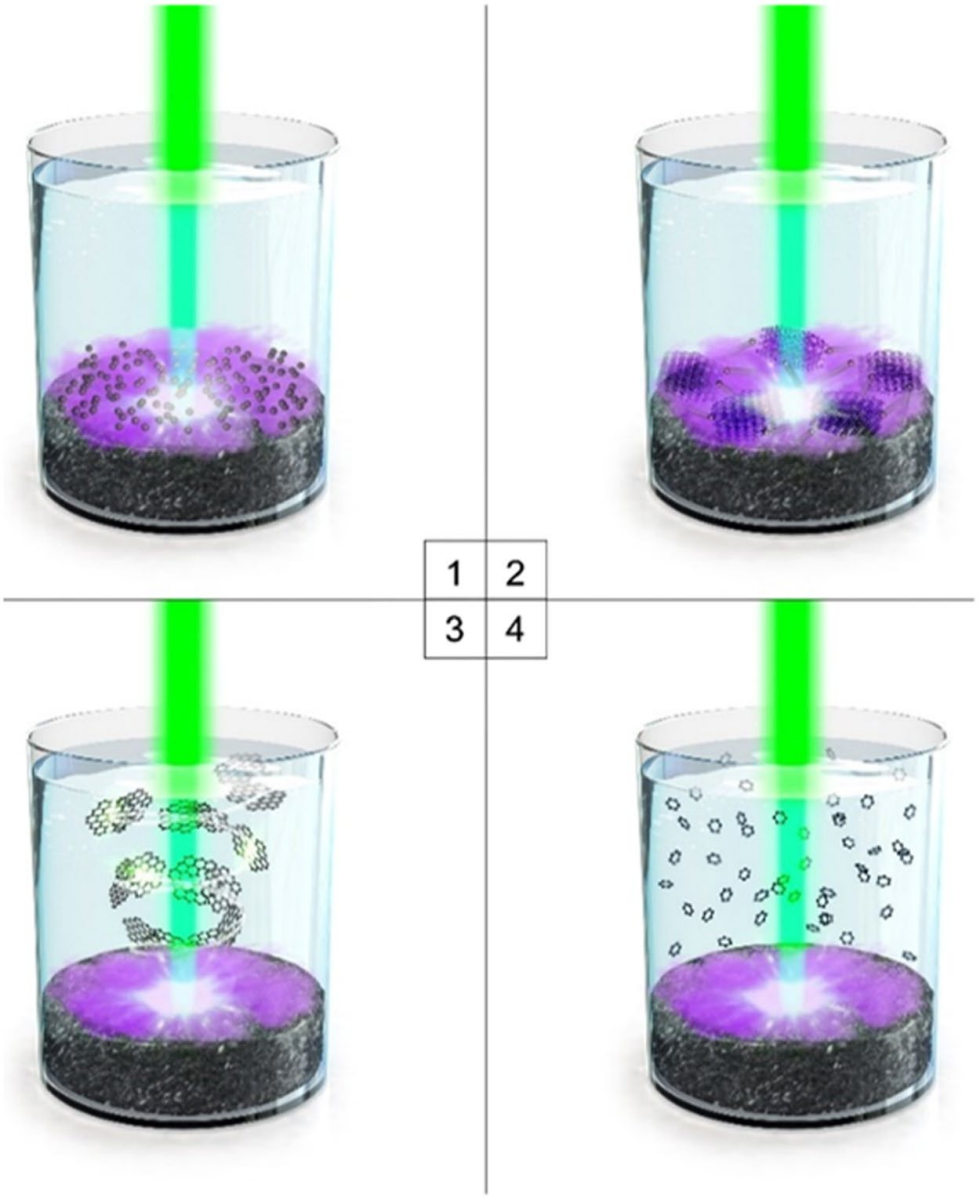
Representative illustration for the possible mechanism of the transform coal to GOQDs by PLAL.

A possible mechanism for the transformation of coal into GOQDs by the PLAL process is proposed in Fig. 2. Coulomb explosions occur at the surface of the solid target during laser-pulse injections. Subsequently, a high-temperature and high-pressure plasma plume forms around the target due to multi-photon absorption ionization^22,23^. A cavitation bubble is generated in the solution as the plasma plume expands and cools down (step 1 in schematic illustration). Carbon clusters in the cavitation bubble that were ablated from the target and have high surface energies tend to aggregate, resulting in layered graphene sheets as the temperature decreases and the internal pressure of the bubble drops to value lowers than those of the surrounding solution (step 2 in schematic illustration)^26,28^. Carbon aggregates prefer to form two-dimensional (2D) structures due to the hexagonal-monolayer structure of pristine carbon. To verify this, *ex-situ* TEM analysis was undertaken to investigate the formation of carbon aggregates from coal. Ten shots of laser (0.1 J each pulse energy) was irradiated to the target of coal and the ablated clusters were collected to be examined by TEM. After ten shots of irradiation, large graphene sheets with 2–3 μm long and 1 μm width were formed, which is consisted of a number of layered graphene sheets with an average size of c.a. 100 nm (Supplementary Fig. S1a–c). HR-TEM image revealed highly crystalline structure of the graphene sheet (Supplementary Fig. S1d). Insets figures are the demonstrate pristine hexagonal structure with well-defined lattice fringe (0.24 nm) of the layered graphene sheet. The layered graphene sheets are further ablated by the injecting laser to form few-nanometer-sized GOQDs (step 3 and 4 in schematic illustration, Supplementary Fig. S1e). A plasma fume can generally be induced at the solid target with laser-power densities in excess of 10^9^ W/cm^2^. A laser source with a power density of 1.2 × 10^9^ W/cm^2^ was used during PLAL in this study; hence, a plasma plume was formed around the coal^22,26^. Control experiments were performed using a laser source with a power density less than 10^9^ W/cm^2^. No plasma plume formed and only bulk graphite-flakes with height of c.a. 6 nm were produced (Supplementary Fig. S2). In addition, the observed variation in GOQD size is possibly ascribable to different cooling rates within the plasma plume (Fig. 1a,d).

**Figure 3:**
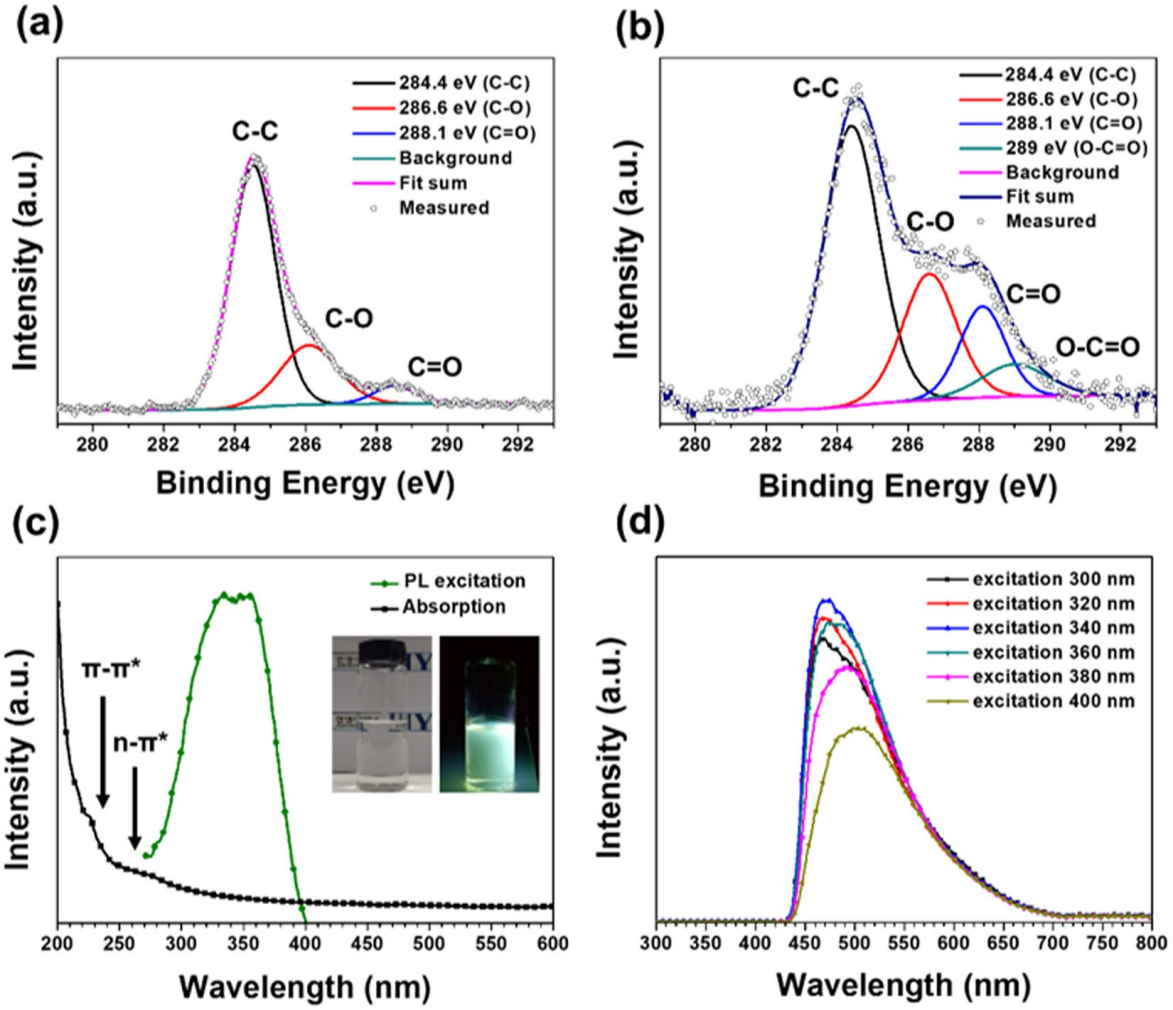
XPS spectra and optical properties of GOQDs. (**a**) High-resolution XPS C1s spectra of coal. (**b**) The XPS C1s spectra of GOQDs. (**c**) UV-vis spectra and PLE spectra of GOQDs. Insets of digital images of GOQDs under excitation of 365 nm. (**d**) PL spectra of GOQDs with the various excitation source.

The GOQDs were subjected to X-ray photoelectron spectroscopy (XPS) in order to investigate their chemical compositions and bindings. The XPS spectra in Supplementary Fig. S3 reveal a dominant graphitic C1s peak at 284.8 eV and an O1s peak at 532.2 eV for both coal and the GOQDs. The C/O ratio observed for the GOQDs was lower than that of coal, which is probably due to the formation of oxygen functional groups. Figure 3a,b display high-resolution C1s spectra of coal and the GOQDs, which can be deconvoluted into three bands with binding energies of 284.4, 286.6, and 288.1 eV that are associated with sp2 aromatic carbons (C=C), epoxy groups (C-O-C), and carboxyl groups (C(O)-O-H), respectively^14,15^. Comparing the C1s spectra, it reveals distinct differences in the carbon chemical environments of coal and the GOQDs. A higher degree of oxidation was observed for the GOQDs compared to coal, as confirmed by the decrease in the calculated sp^2^ and sp^3^ ratio (Supplementary Table S3). A larger fraction of oxygen functional groups facilitates the easy dispersion of the GOQDs in aqueous solutions, which is an essential property for bioimaging applications^16,32^.

We investigated the optical properties of GOQDs by ultraviolet-visible (UV-Vis) absorption and photoluminescence-excitation (PLE) spectroscopy. The PLE spectrum of the GOQDs was measured at the emission of 480 nm as shown in Fig. 3c. The PLE spectrum shows the distinct one peak centered at 340 nm, which is similar to the reported PLE peak of GOQDs prepared by chemical exfoliation method^18^. The insets of Fig. 3c shows the GOQDs under the UV irradiation with excitation wavelength of 360 nm. The distinct green emission of GOQDs is clearly represented. Figure 3d shows the PL spectra of GOQDs with different excitation wavelengths. One can find that the PL peak gradually red shifts when the excitation wavelength changed from 300 to 400 nm. Also, as the excitation wavelength changed from 300 to 340 nm, the peak intensity gradually increased. However, peak intensity of the emission was abruptly decreased when excitation wavelength exceeded 340 nm (Fig. 3d). The excitation wavelength dependent PL is attributable to surface functional groups (e.g. surface defects, OH^−^) that exhibit different energy levels acting as trap states for excitons^10–12,19–21^. To further understand the PL properties, we investigated the TRPL (Time-Resolved photoluminescence) lifetime of the GOQDs (Fig. S4). The Fluorescence lifetimes of GOQDs are recorded at 550 nm, and the excitation wavelength is 370 nm which is provided by a laser diode. The fluorescence decay curve is fitted with triexponential function. The lifetime of GOQDs are τ1 = 2.4 ns, τ2 = 0.5 ns and τ3 = 5.9 ns were observed. Typically, the emission that originates from defect states shows a longer recombination lifetime than that intrinsic states, as often observed in typical GOQDs^18^. Consequently, among the three lifetimes, one is due to intrinsic state (τ2 = 0.5 ns) and the other two (τ1 = 2.4 ns, τ3 = 5.9 ns) are due to the presence of oxygen functional groups on the surface of GOQDs. Liu *et al*. described the PL mechanism of GOQDs through defect-state emissions related to oxygen functional groups^18^. Since our GOQDs have high concentrations of oxygen surface functional groups, as revealed by XPS (Fig. 3b), their observed PL behavior can also be attributed to various defect states caused by surface oxygen functional groups. Supplementary Fig. S5 summarizes the possible PL mechanism of GOQDs. The GOQDs consists of numerous disorder-induced defect states within the π-π* gap. Electrons are excited to energy level of the lowest unoccupied molecular orbital (LUMO) band, and it moves to the defect state by non-radiative relaxation determining the PL properties of GOQDs^11,18^. Furthermore, the GOQDs exhibit typical absorption peaks centered at around 220 and 270 nm (Fig. 3c). The extinction mass coefficient of GOQDs was calculated using the below equation,

1 $${\rm{A}}={\rm{\varepsilon }}\mathrm{ic}$$

where c is the concentration of GOQDs in solvent (0.1 mg/mL), A is the absorption cross section of GOQDs at wavelength of 230 nm (0.8), and i is the thickness of the cuvette (1 cm). The result shows that our GOQDs have a 1.2 × 10^2^ M^−1^cm^−1^ extinction coefficient in the UV region. Based on literature reports, these peaks are attributed to π-electron transitions in oxygen-containing GOQDs. The 220 nm peak corresponds to the π-π* transitions of aromatic C=C bonds, while that at 270 nm corresponds to n-π* transitions of C=O bonds. In addition, our GOQDs exhibit an absorption peak with a long (up to 600 nm) tail, which reveals the existence of surface defect sites in these GOQDs^18,19^. Quantum yield (QY) was calculated for the GOQDs. Details for QY calculations are summarized in the supplementary information. The result indicates that the QY of GOQDs is about 0.8–0.9%, which is comparable to the QYs of other GOQDs prepared by PLA method (Supplementary Table S4). In addition, the optical- and photo-stabilities of the GOQDs were examined. GOQD photostability was investigated by comparing the PL emission spectra of as-prepared samples and samples aged for 1–2 month (Supplementary Fig. S6). The results reveal negligible changes in PL intensity, even after 2 months; clearly our GOQDs are highly photostable. The photo-stability was tested under the continuous UV lamp illumination with a power of 250 W for different durations. As shown in Supplementary Fig. S7, there were negligible changes in PL intensities for the GOQDs during 180 min. The high QY, excellent photostability and brightness of emitting green PL make our GOQDs suitable for the bioimaging applications.

**Figure 4:**
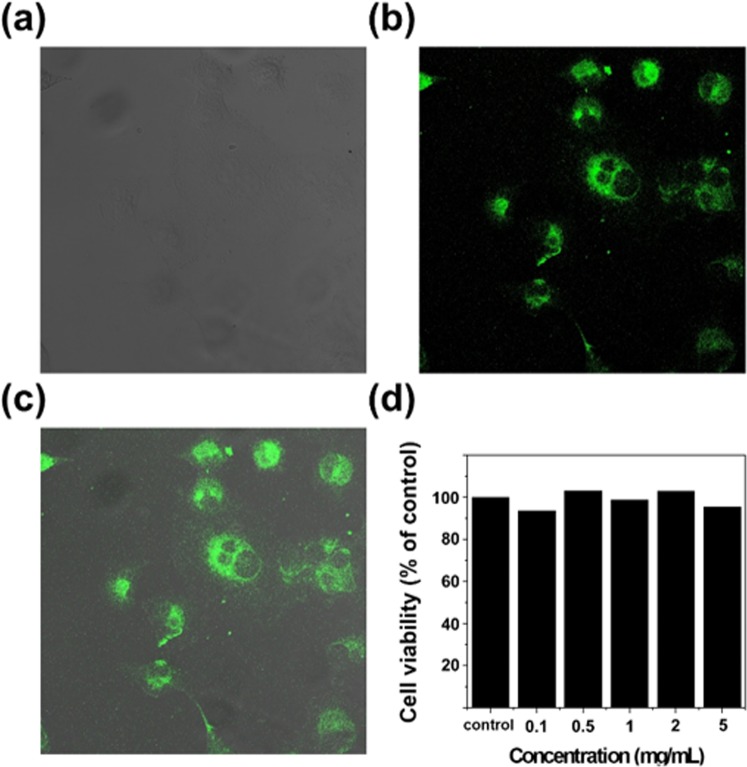
Fluorescence images of human pancreatic cancer cell PanC-1 after incubation with GOQDs for 24 h. (**a**) Phase contrast image of PanC-1 cells. (**b**) Agglomerated GOQDs surrounding each nucleus. (**c**) The overlay high contrast images of GOQDs. (**d**) Cell viability assay with PanC-1 cell with different concentration of GOQDs.

To assess the prepared GOQDs for potential bioimaging applications, GOQD cytotoxicity was investigated using the 3-(4,5-dimethylthiazol-2-yl)-2,5 diphenyltetrazolium bromide (MTT) assay (see the Supporting Information for details). As shown in Fig. 4a–c, PanC-1 cells effectively retain their original morphologies and exhibited green PL upon incubation with the GOQDs. Figure 4d shows that PanC-1 cell viability remained greater than 85% as the GOQD concentration was increased from 0.1 to 5 mg mL^−1^, which indicates that our GOQDs are highly biocompatible and exhibit low cytotoxicity. These results highlight the practical use of our GOQDs as PL probes for bioimaging and related applications.

In conclusion, GOQDs have been successfully prepared through a facile, low cost and environmental friendly PLAL process using earth-abundant and low-cost coal as carbon source. Ablated carbon clusters from the coal can be completely transformed to GOQDs in 5 min. The mechanism for the formation of GOQDs was revealed by *ex-situ* TEM analysis. The newly developed PLAL process in this work has major advantages as follows: (1) no need for the use of strong acidic or basic solutions and thus post purifying process is not required. (2) Fast, facile, environmentally friendly one-step process for prepared GOQDs. (3) GOQDs with excellent photostability, optoelectronic properties, low toxicity and biocompatibility can be synthesized, all of which allows our GOQDs can be applied to PL imaging *in vitro*.

## Methods

### Reagents and instruments

Coal activated granular was purchased from DAEJUNG (republic of korea) and High purity ethanol was purchased from Sigma Aldrich. HR-TEM images were taken using a 2100 F field emission gun TEM (JEM 2100 F, USA, 200 KV) for GOQDs samples. XPS spectra were recorded for the both samples using VG ESCALAB 220i (Thermo scientific, USA). XPS survey and high resolution scans were performed with the pass energies of 100 eV and 20 eV, respectively. X-ray beam size was approximately 100 μm. GOQDs samples for XPS measurement were prepared via spin coating technique. Silicon (Si) substrate was used for spin coating. Rotation speed was adjusted to 3,000 rpm. The samples were dried at room temperature for 2 hrs before the measurement. Room temperature PL spectra of GOQDs were collected using a photoluminescence spectrophotometer (PerkinElmer, LS55 with 100 mW laser diode, USA). UV-vis absorption spectra were carried out with Lamda 650 S UV-vis spectrophotometer (PerkinElmer).

### Preparation of GOQDs

GOQDs were fabricated by the simple and facile PLAL process using coal in high-purity ethanol. In a typical process, 1 g of coal (purity of up to 90%) was fixed in the bottom of a glass vessel containing 50 mL of high-purity ethanol. A Q-switch ND:YAG laser system was employed at room temperature and in the air. The coal suspension, which forms a vertical water column, was ablated by a horizontal pulsed laser beam (355 nm, third harmonic, 10 ns pulse width) at a repetition rate of 20 Hz. The pulsed laser beam was focused to a diameter of around 1 mm with an ablation energy of 0.1 J.

### Cellular imaging and cytotoxicity assays

The living PanC-1 cell were grow in DMEM (Dulbecco’s Modified Eagle’s Medium) supplemented with 10% FBS (fetal bovine serum) and incubated at 37 °C in 5% CO2 atmosphere. The cell was then incubated with GOQDs (1 mg mL^−1^) in medium (2 mL) for 1 h at 37 °C and washing with phosphate buffered saline (PBS) three times to remove the extracellular GOQDs. After that, the cell fluorescence images were acquired using an oil dipping objective (×100) on a confocal laser scanning fluorescence microscope setup (leica Model TCS SP5). The cytotoxicity of GOQDs was examined by MTT assay. First, PanC-1 cells were seeded in 96-well plates. After incubating for 24 h, the medium was then replaced by the culture solution containing GOQDs with various concentrations (0.1, 0.5, 1, 2 and 5 mg ml^−1^), and the cells were continued to be incubated for another 24 hr. After that, the cells were washed three times with PBS, and then, freshly prepared MTT (0.5 mg ml^−1^) solution was added to each well. Finally, the MTT medium solution was carefully removed after 4 h incubation, and dimethyl sulfoxide (DMSO) was then added into each well. The plate was gently shaken for 10 min at room temperature to dissolve all precipitates, and the absorbance of MTT at 570 nm was monitored in a spectrophotometer.

## Supplementary information

Supporting Information
