# Publisher Correction: Graphene Oxide Quantum Dots Derived from Coal for Bioimaging: Facile and Green Approach

**DOI:** 10.1038/s41598-020-60499-0

**Published:** 2020-04-29

**Authors:** Sukhyun Kang, Kang Min Kim, kyunghwan Jung, Yong Son, Sungwook Mhin, Jeong Ho Ryu, Kwang Bo Shim, Byoungsoo Lee, HyukSu Han, Taeseup Song

**Affiliations:** 1 Korea Institute of Industrial Technology, Gwahakdanji-ro 137-41, Gangwond-do, 25440 Republic of Korea; 2 Department of Materials Science and Engineering, Hanyang University, Seoul, 133-791 South Korea; 3 Korea Institute of Industrial Technology, 113-58, Seohaean-ro, Siheung-si, Gyeonggi-do, 15014 Republic of Korea; 4 Department of Materials Science and Engineering, Korea National University of Transportation 50 Daehak-ro, Chungju-si, Chungbuk 380-702 Republic of Korea; 5 Department of Materials Science and Engineering, Hongik University, Sejong 30016 South Korea; 6 Department of Energy Engineering, Hanyang University, Seoul, 133-791 South Korea

Correction to: *Scientific Reports* 10.1038/s41598-018-37479-6, published online 11 March 2019

In the original version of this Article, there were errors in the Affiliations.

Korea Institute of Industrial Technology, School of Materials, Science and Engineering, Hongik University, Sejong, 339-701, Republic of Korea

now reads:

Korea Institute of Industrial Technology, Gwahakdanji-ro 137-41, Gangwond-do, 25440, Republic of Korea.

Department of Energy Engineering, Hanyang Universiy, Seoul, 133-791, South Korea

now reads:

Department of Energy Engineering, Hanyang University, Seoul, 133-791, South Korea

Additionally, an affiliation for HyukSu Han was omitted. The correct affiliations for HyukSu Han are listed below:

Korea Institute of Industrial Technology, Gwahakdanji-ro 137-41, Gangwond-do, 25440, Republic of Korea.

Department of Materials Science and Engineering, Hongik University, Sejong, 30016, South Korea.

These errors have now been corrected in the HTML and PDF versions of this Article, and in the accompanying Supplemental Material.

