# Supplementary material for: Graphene Oxide Quantum Dots Derived from Coal for Bioimaging: Facile and Green Approach
Source: Sci Rep. 2019 Mar 11;9:4101. doi: 10.1038/s41598-018-37479-6 (PMC6411918; doi:10.1038/s41598-018-37479-6)
Supplement: Supplementary file 1 — Supporting Information [file 41598_2018_37479_MOESM1_ESM.docx]

***Supporting Information (SI) for:***

**Graphene Oxide Quantum Dots Derived from Coal for Bioimaging: Facile and Green Approach**

Sukhyun Kang^1,2,§^, Kang Min Kim^1,§,*^, Kyunghwan jung^1^, Yong Son^3^, Sungwook Mhin^3^, Jeong Ho Ryu^4^, Kwang Bo Shim^2^, HyukSu Han^1,*^, Taeseup Song^5,*^

^1^Korea Institute of Industrial Technology, Gwahakdanji-ro 137-41, Gangwond-do, 25440, Republic of Korea

^2^Department of Materials Science and Engineering, Hanyang University, Hanyang Universiy, Seoul 133-791, South Korea.

^3^Korea Institute of Industrial Technology, 113-58, Seohaean-ro, Siheung-si, Gyeonggi-do, 15014, Republic of Korea

^4^Department of Materials Science and Engineering, Korea National University of Transportation 50 Daehak-ro, Chungju-si, Chungbuk 380-702, Republic of Korea

^5^Department of Energy Engineering, Hanyang Universiy, Seoul 133-791, South Korea.

^§^These authors contributed equality to this work. ^*^Correspondence and requests for materials should be addressed to K.M.K (email: [kmkim@kitech.re.kr](mailto:kmkim@kitech.re.kr)) or H.H (email: [hhan@kitech.re.kr](mailto:hhan@kitech.re.kr)) or T.S (email: tssong@hanyang.ac.kr)

**Quantum yield (QY) measurement**

Quinine sulfate in water (QY=0.58) and fluorescein in ethanol (QY = 0.79) were chosen as standards for measuring QY of GOQDs excited. The QY of GOQDs (in water) was calculated according to :

$Ф_{x}$ = $Ф_{\mathrm{st}}{(I}_{x}/I_{\mathrm{st}}){(\eta}_{x}^{2}/\eta_{\mathrm{st}}^{2}) {(A}_{\mathrm{st}}/A_{x})$

where Ф is the quantum yield I is the measured integrated emission intensity, η is the refractive index of the solvent, and A is the optical density. The subscript “st” refers to standard with known QY and “x” for the sample. In order to minimize re-absorption effects, absorption in the 10 mm fluorescence cuvette was kept below 0.10 at the excitation wavelength of 340 nm. The QYs of GOQDs were measured to be 0.8 ~ 0.9% under excitation wavelength of 340 nm. The excitation wavelength used in the QY measurements gave the highest PL emission in the PLE spectra (Figure 2d). The QY value was compared to QYs data reported from elsewhere (Supplementary Table S4).


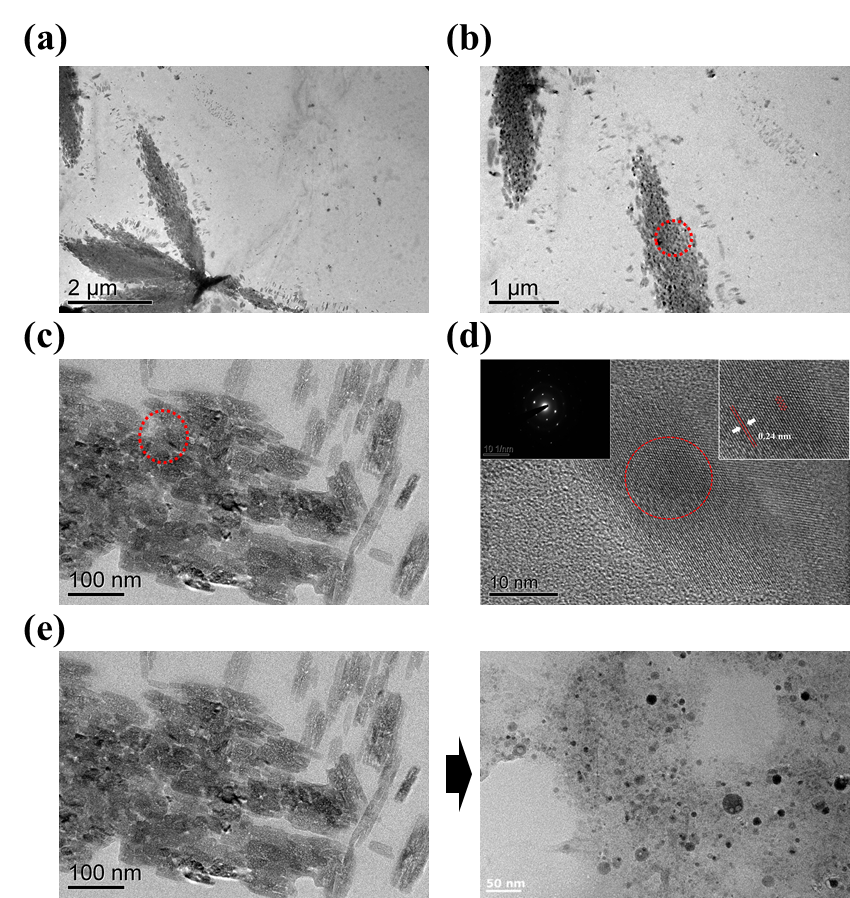


**Supplementary Figure S1.** (a-d) TEM and HR-TEM images of large graphene sheet by the ten shots of laser ablation (e) HR-TEM images of transform graphene sheet to GOQDs are further ablation.


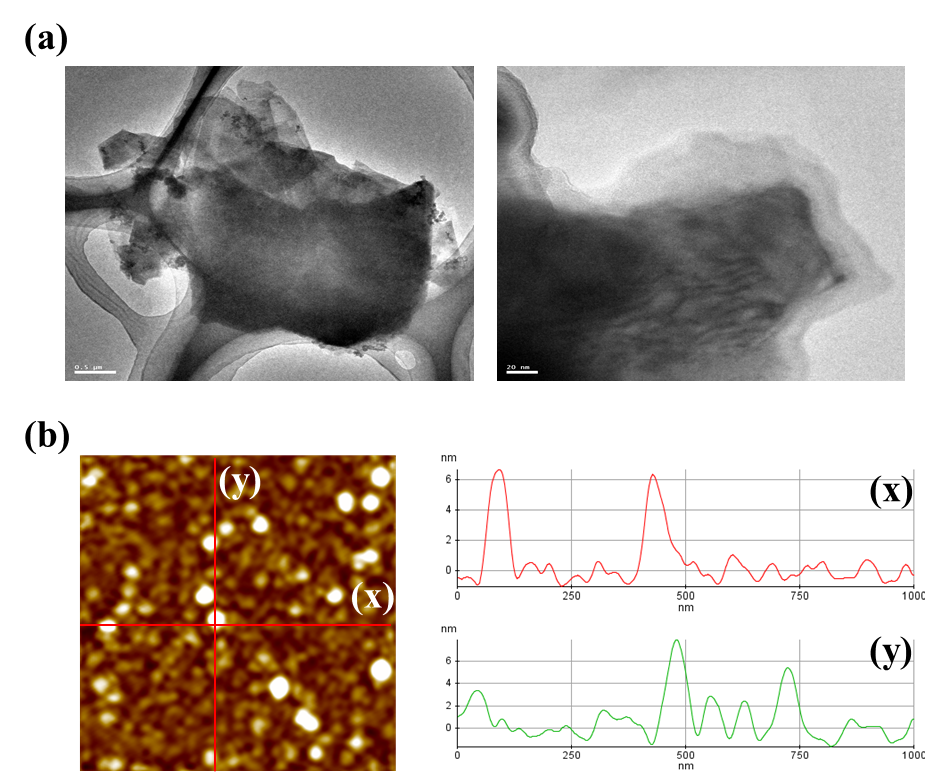


**Supplementary Figure S2.** TEM and AFM characterization of coal for control experimental (a) TEM images of coal after laser ablation (laser power 0.05J). (b) AFM images and height histogram of coal after laser ablation.


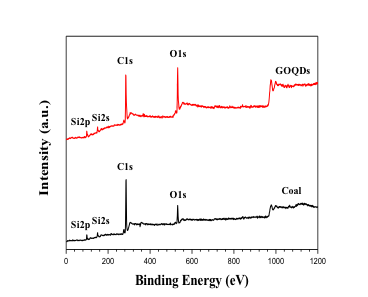


**Supplementary Figure S3** XPS survey spectrum of coal and GOQDs.

| 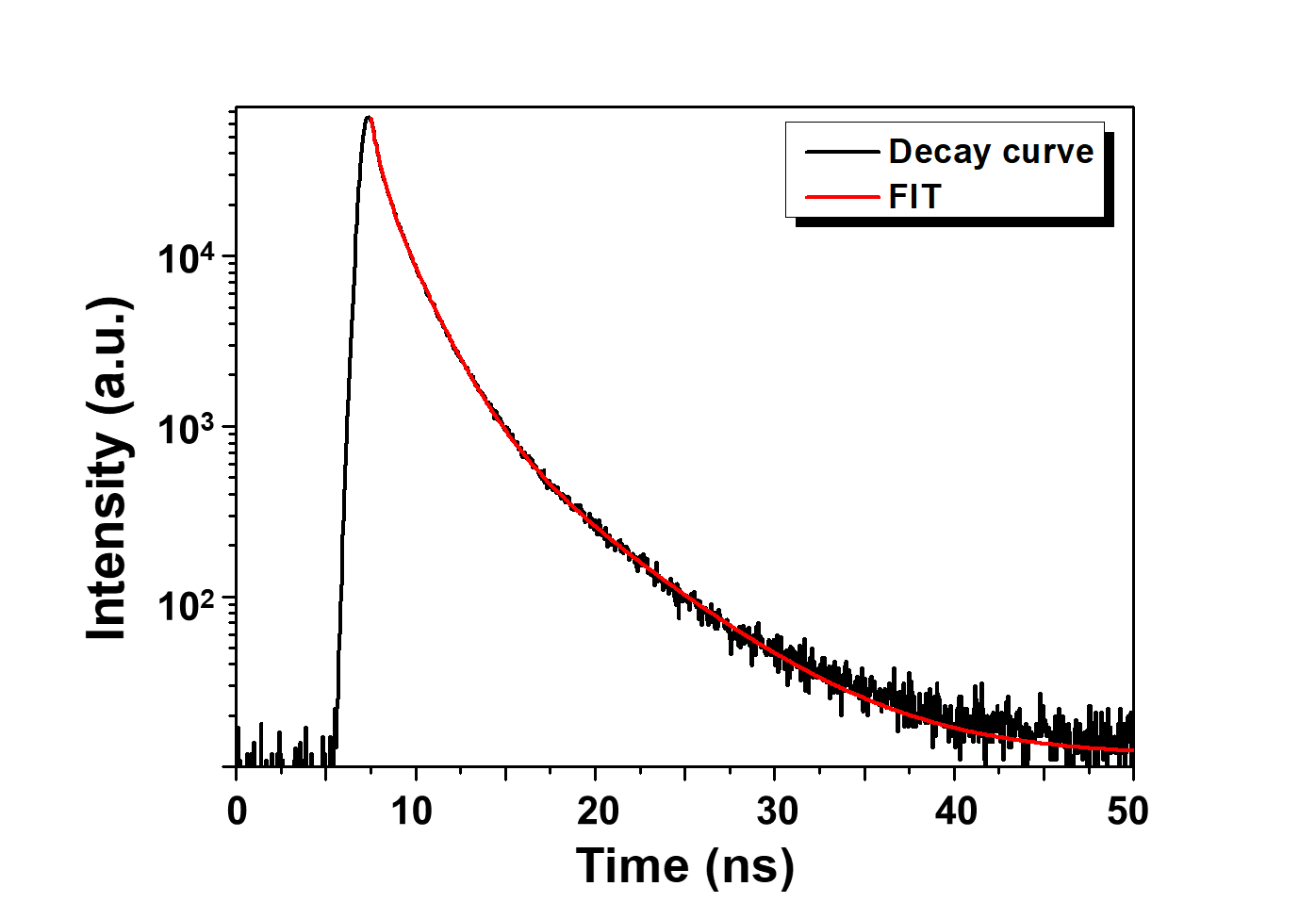 |
| --- |
| **Supplementary Figure S4.** Time-Resolved photoluminescence spectra of GOQDs. |


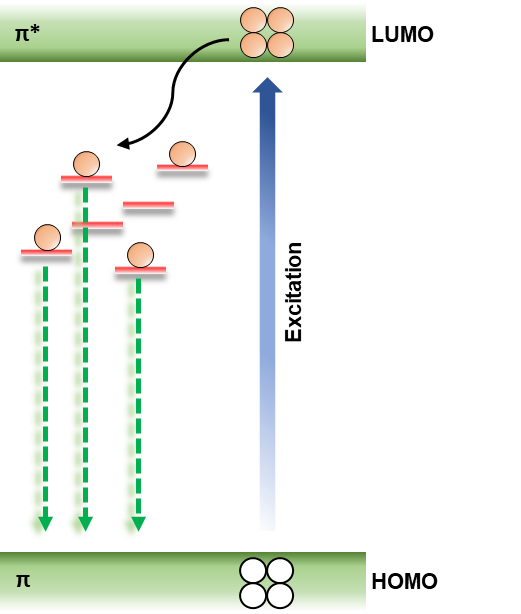


**Supplementary Figure S5.** Schematic diagram for the PL mechanism of GOQDs.


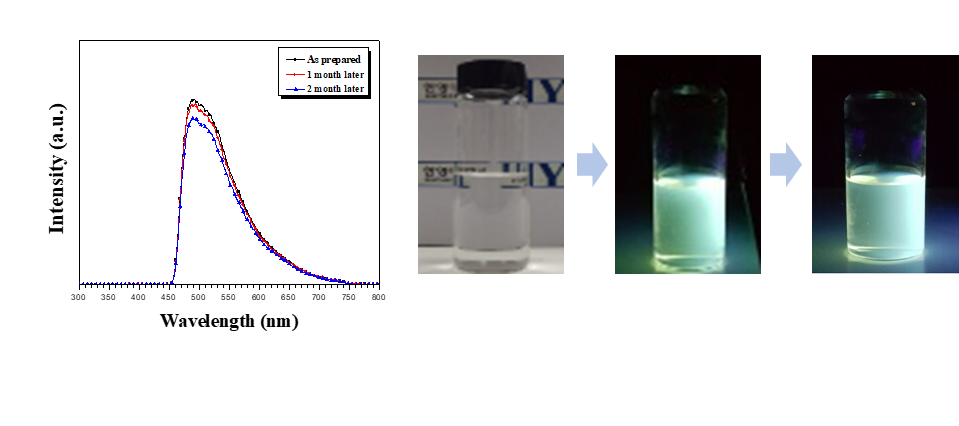


**Supplementary Figure S6.** Time dependent PL emission spectra and digital images (under 365 nm excitation.

2 month later

1 month later

As-prepared


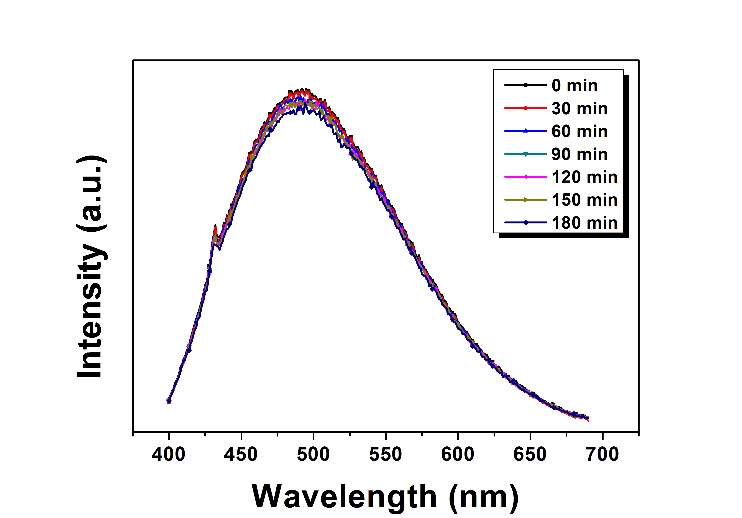


**Supplementary Figure S7. .** Dependence of PL intensity on UV excitation time for GOQDs.

**Supplementary Table S1 The world’s major regions for coal production and consumption. [S1]**

| Region | Production  (Million tonnes) | Consumption  (Million tonnes) |
| --- | --- | --- |
| Asia | 2702.3 | 2780 |
| North America | 407.9 | 363.8 |
| Europe and Eurasia | 164.6 | 296.4 |

**Supplementary Table S2. Raw materials prices for prepared of GQDs. [S2]**

| Raw materials | Price | Ref. |
| --- | --- | --- |
| Natural graphite | 113 g / 21.57 $ | 17 |
| Graphene oxide | 1 g / 161.32 $ | 26 |
| Carbon nanofiber | 25 g / 182.71 $ | 21 |
| MWCNTs | 1 g / 178.25 $ | 15 |
| Coal | 1ton / 12.35 $ | This work |

**Supplementary Table S3. XPS survey of coal and GOQDs.**

|  | Peak  Binding Energy | Coal | GOQDs |
| --- | --- | --- | --- |
| C=C and C-C (%) | 284.4 | 82.2 | 55.87 |
| Hydroxyl, Carboxyl (%) | 286-289 | 17.8 | 44.13 |

**Supplementary Table S4. A summary of QY values via PLA method.**

| Subclassification | Starting  Materials | Size (nm) | Color | Quantum yield (%) | Ref. |
| --- | --- | --- | --- | --- | --- |
| Pulsed Laser | MWCNTs | 2-5 | Green, blue | 0.8 | 19 |
| Pulsed Laser | Nickel oxide  Benzene | 2-5 | Blue | 0.055 | S3 |
| Pulsed Laser | Graphene  Oxide | 5-30 | Green, blue | 1.8 | S4 |
| Pulsed Laser | Coal | 5-20 | Green | 0.8-0.9 | This work |

**References.**

[S1] BP global, BP Statistic Energy Review of World Energy June 2017 (June 2017; http://www.bp.com/liveassets/bp_internet/globalbp/globalbp_uk_english/reports_and_publicatio ns/statistical_energy_review_2011/STAGING/local_assets/pdf/coal_section_2017.pdf).

[S2] U.S. Energy Information Administration, Coal News and Markets (April 8, 2017; <http://www.eia.gov/coal/news_markets/>).

[S3] Khaled, H. et al. Luminescent graphene quantum dots fabricated by pulsed laser synthesis. *Carbon* 64, 341-350 (2013).

[S4] T.N. Lin, et al. Laser- ablation production of graphene oxide nanostructures: from ribbons to quantum dots. *Nanoscale* 7, 2708 (2015)
